# Factors associated with change in self-reported physical activity in the very old: The Newcastle 85+ study

**DOI:** 10.1371/journal.pone.0218881

**Published:** 2019-07-16

**Authors:** Antoneta Granic, Karen Davies, Richard M. Dodds, Rachel Duncan, Germaine Uwimpuhwe, Eduwin Pakpahan, Siân Robinson, Avan A. Sayer

**Affiliations:** 1 AGE Research Group, Institute of Neuroscience, Newcastle University, Newcastle upon Tyne, United Kingdom; 2 NIHR Newcastle Biomedical Research Centre, Newcastle upon Tyne Hospitals NHS Foundation Trust and Newcastle University, Newcastle upon Tyne, United Kingdom; 3 Newcastle University Institute for Ageing, Newcastle upon Tyne, United Kingdom; 4 Institute of Health & Society, Newcastle University, Newcastle upon Tyne, United Kingdom; Anglia Ruskin University, UNITED KINGDOM

## Abstract

**Background:**

Higher physical activity (PA) has been linked to better health and functioning. Trajectories of PA and associated factors have been studied in older adults aged ≥65, but less is known about influences on PA change in the very old (aged ≥85).

**Objective:**

To investigate factors associated with self-reported PA and PA change over time in very old adults.

**Methods:**

845 participants in the Newcastle 85+ Study were followed for health and functioning at 1.5-, 3-, and 5-year follow-up (wave 2 to 4). PA scores (range 0–18) and PA levels (low (PA scores 0–1), medium (2–6) and high (7–18)) were determined using a purpose-designed PA questionnaire. We used linear mixed models (LMM) to investigate factors associated with 5-year change in PA scores.

**Results:**

Overall, men had higher mean PA scores than women (up to 2.27 points). The highest proportion of participants (42–48%) had medium levels of PA across the waves. Although most experienced decline—stability in moderate and increases in high PA levels were also observed. The fully adjusted LMM revealed a curvilinear annual decline in PA scores of 0.52 (0.13) (β (SE), p<0.001), which decelerated by 0.07 (0.02) points (p<0.01) over time. The factors associated with low PA scores at baseline were female gender, higher waist-hip ratio, and no alcohol intake. Better self-rated and cognitive health and having fewer diseases were associated with higher PA scores. None were associated with the rate of change in PA over time.

**Conclusion:**

We observed a curvilinear trend and deceleration in PA scores decline in the very old. Men and those in better health and who drank alcohol were more physically active at baseline. None of the factors were associated with the rate of PA decline. Investigating those who maintain or increase levels of PA may inform interventions for at risk groups with PA decline.

## Introduction

The health benefits of physical activity (PA) as a modifiable lifestyle factor across the life course have been firmly established. The latest 2018 US Physical Activity Guidelines Advisory Committee Report [1] for older adults aged 65 and over (65+) has highlighted new evidence that even a sporadic accumulation of 150 minutes (2.5 hours) a week of moderate-to-vigorous intensity PA in bouts of any duration reduces the risk of all-cause mortality, cardio-metabolic diseases, brain and mood disorders, incidence of falls, and improves physical functioning [1]. The 2018 follow-up draft report [2] of the UK Chief Medical Officer’s Physical Activity Guidelines for Older Adults (2011) [3] has provided further Level 1 evidence about the benefits of PA in later life. Older adults engaging in moderate intensity PA have lower incidence of disability and severity of symptoms of several age-related diseases. Those who are active daily (measured by frequency of trips from home) have reduced mortality and incident disease [2]. Several international PA guidelines have recognised the need for a specific set of recommendations for healthy older adults aged 65+, versus those in poor health, around the paradigm of ‘maintenance of function’ [4–8], but none have proposed PA recommendations by age group. Due to the heterogeneity of ageing experience across older ages, optimal PA levels for health and functioning of the young-old (aged 65–74) compared with the very old (aged 85+) may differ in frequency, intensity and duration.

Despite the numerous health benefits of PA, the percentages of older adults who meet the recommendations for PA (assessed subjectively using PA questionnaires or objectively by accelerometry) are low, particularly in women. For example, only 10% of women and 15% of men aged 70–93 in the British Regional Heart Study and the British Women’s Heart Health Study [9], and 18% of individuals aged 75+ in the Irish Longitudinal Study of Ageing [10] met the recommended 2.5 hr of moderate PA/week. Decline in PA becomes more marked with advanced age [3], especially after the age 70 and over [11,12]. Cross-sectional normative data from the National Health and Nutrition Examination survey has shown a substantial decline in average steps/day from 2,500–4200 in women aged 70+ to about 500–900 steps in those aged 85+ (the very old) [11]. Identifying factors that influence decline in PA may help in designing strategies aimed to preserve functional status and independence in older adults [13].

A number of studies have examined long-term changes in self-reported PA in older adults aged 65+ (e.g. [14–23]), but few included individuals aged 85+ (e.g. [14,19,22]), and none are focused on this age group alone. Aside from advanced age, factors identified in previous studies to be associated with low PA in older adults were female gender, lower education or social class, poor self-rated health, chronic diseases, obesity, higher BMI or waist-hip ratio, depression, cognitive impairment, functional limitations, low self-efficacy, perceived stress, smoking, not drinking alcohol, and small social networks [14,15,17,18,20–26]. Currently it is not known how PA changes over time in later life, and whether these factors are associated with PA in the very old. The very old are the fastest growing age group in many societies [27,28] and are at highest risk of ill health and functional decline [29,30], which may be exacerbated further by inactivity and depleted physiological reserve [31]. Cohort studies of the very old are invaluable in understanding changes in health and functioning in more life-like settings, and may provide information relevant at the population level [32].

Therefore, using baseline and prospective data from a cohort study of the very old, the Newcastle 85+ Study, we aimed to describe factors related to self-reported PA and to investigate factors associated with change in PA over a 5-year follow-up.

## Methods

### Study cohort

We included 845 participants (62% (526) women; mean age 85.49 (standard deviation (SD) = 0.44)) from the Newcastle 85+ Study, a longitudinal population-based study of individuals born in 1921 and residing in Newcastle and North Tyneside, United Kingdom, who had a complete multidimensional health assessment and general practice records review (GPRR) at baseline (2006/07, wave 1). The study details have been published previously [33,34] and are available at http://research.ncl.ac.uk/85plus/. Participants were followed up for an assessment of health and functioning (including self-reported physical activity, PA) at 18 (1.5 years; wave 2), 36 (3 years; wave 3), and 60 months (5 years; wave 4) by research nurses at their usual place of residence.

The study was approved by the Newcastle & North Tyneside Local Research Ethics Committee 1 (Ref: 06/Q0905/2). Signed informed consent was obtained from each participant. For those lacking capacity, the consent was sought from their consultee or carer.

### Physical activity

A PA questionnaire (PAQ) was purposely designed based on the results from the Newcastle 85+ pilot study [33,35] and used at each wave. Participants were asked about type and amount of PA involved in their daily lives which was either highly energetic, moderately energetic, or mildly energetic (Table 1).

**Table 1:** Examples of physical activities in ‘very energetic’, ‘moderately energetic’ and ‘mildly energetic’ category.

Very energetic	Moderately energetic	Mildly energetic
Swimming	Moderate gardening	Light gardening
Cycling	Cleaning the car	Bowling
Running	Heavy housework (cleaning windows, scrubbing floors)	Light housework ‘Do it yourself’ (DIY)
Heavy gardening (digging with a spade, manually mowing the lawn)	Walking at moderate pace	
	Floor or stretch exercise	

The frequencies of each activity intensity were recorded as: (a) three or more times a week (score of 3); (b) once or twice a week (score of 2); (c) once, twice or three times per month (score of 1), and (d) hardly ever or never (score of 0) (Table A in S1 File). From these responses, we obtained three outcome variables: very energetic, moderately energetic and mildly energetic activities (score range of 0–3 for each). To derive an overall PA score we used the following formula: (3×very energetic activities score) + (2×moderately energetic activities score) + (1×mildly energetic activities score). The overall PA score ranged from 0–18, and were used as continuous variable and further categorised into three levels (low (0–1); medium (2–6); and high (7–18)) PA [35]. PAQ was repeated at wave 2 to wave 4 (1.5, 3 and 5 years from baseline). At follow-up, 629 (wave 2), 483 (wave 3) and 344 (wave 4) participants had PA score and established PA levels (Table B in S1 File).

The PAQ previously showed convergent validity with objective measures of PA (5–7 days accelerometry), including activities classified as sedentary, activities of daily living (ADL) and walking [35]. Briefly, 337 (70%) participants who completed the PAQ in wave 3 wore a triaxial accelerometer (GENEA, Unilever, UK) on the right wrist for a period of 5–7 days. Self-reported PA was correlated with objective measures of PA (Spearman’s ρ = 0.10–0.52) and these self-reported scores were significantly different when low, moderate and high self-reported PA categories were compared (all p<0.001) [35].

### Other measures and potential confounders

In the multivariable analyses, we used the following determinants from the literature that had been found to be associated with changes in PA in older adults (e.g. [21–26]). Sociodemographic variables included sex and occupational class (higher managerial, administrative and professional occupations / intermediate occupations / routine and manual occupations) coded to the National Statistics Socio-economic Classification System. Anthropometric measurements included waist-hip ratio (continuous). Lifestyle variables were: energy consumed from foods (in megajoules (MJ), continuous), smoking status (current smoker/former smoker/never smoker), current alcohol intake (yes / no). Health-related factors were: cognitive status (Standardised Mini-Mental State Examination (SMMSE) categorised as cognitively impaired (score 0–25), and normal (score 26–30)); depressive symptoms (Geriatric Depression Scale categorised as normal (score 0–5), mild (score 6–7), and severe (score 8–15) depressive symptoms); self-rated health (excellent or very good / good / fair or poor), number of chronic diseases (0–1 / 2 / 3 and more medically diagnosed diseases, from GPRR), number of prescribed medications (0–2 / 3–4 / 5 and more), and having generalised pain in the past month lasting a day or more (yes / no). All confounders included in the analyses were assessed at baseline.

### Statistics

#### Descriptive statistics

We used means and standard deviations (SD) to describe continuous variables, and frequency (n) and percentages (%) for categorical variables. We employed the Shapiro-Wilk test to check for normality assumption, and Levene test for constant variance. For non-normally distributed data we used Kruskal-Wallis test, Gamma test for ordinal variables, and Fisher exact test or Chi-square test for categorical variables to determine the differences across three PA levels. In all analyses, we used α ≤0.05 for statistical significance.

#### Change in physical activity scores over 5-year follow-up

We accounted for the longitudinal nature of data by fitting linear mixed models (LMM) for PA scores (continuous) [36,37]. LLM (growth curve modelling) has several advantages compared to other analyses of repeated observations (e.g. General Linear Model), especially for the measurement occasions nested within individuals which include missing data. LLM allows for a simultaneous examination of within-individual change over time (defined as Level 1), and differences between individuals (defined as Level 2) by examining the relationship between predictors and the shape of individual growth trajectory.

We used restricted maximum likelihood (RML), and unstructured variance component (UN) to generate parameter estimates (β regression coefficients) with standard errors (SE). We investigated linear and quadratic effects of time (continuous) on PA score over the 5-year follow-up (Level 1), and adjusted for potential confounders (Level 2) that were significantly associated with PA levels at baseline or were reported in the literature. Model 1 included ‘time’ (to assess for linear change in PA scores over 5 years), and ‘time^2^’ (time × time; to test for the acceleration or decelerations of change in PA scores over time), and sociodemographic variables (sex and occupational class). Model 2 was additionally adjusted for health-related variables (waist-hip ratio, cognitive status, depressive symptoms, self-rated health, number of chronic diseases, number of prescribed medications, and generalised pain in the past month). Model 3 was further adjusted for lifestyle factors (energy from foods, alcohol intake, and smoking). All predictors were treated as time-invariant variables.

We used the likelihood ratio (LR) test to select the appropriate time parameters. We chose models with the lowest goodness of fit statistics (AIC) and fewest parameters. All models included random intercepts and slopes. β coefficients for ‘time’ and ‘time^2’^ represent the change in PA score over time for the whole population (i.e., an average loss / gain at each time point, and an average change in the rate of change in PA score). β coefficients for confounders show the main cross-sectional effect of each confounder on the overall PA score.

#### Sensitivity and supplementary analysis

Frequencies and percentages of participants reporting very energetic, moderately energetic and mildly energetic activities across the waves and differences between men and women were assessed using Chi-square tests. The prevalence of individual diseases (yes / no) by PA levels from cardiovascular (hypertension, schematic, heart fail, peripheral vascular, atrial fibrillation or flutter, cerebrovascular), osteoarthritis-related, inflammatory arthritis, osteoporosis, chronic obstructive pulmonary disease (COPD), asthma, thyroid, diabetes (type I, II and unspecified), cancer (excluding skin carcinoma and solar keratosis in the past 5 years), and renal impairment (according to Chronic Kidney Disease Epidemiology Collaboration equation) were analysed using the method described in the Descriptive statistics section (Table C in S1 File).

The LMM were additionally adjusted for interaction terms between significant determinants and time, including time × sex to determine whether the rate of change in PA scores over time varied between men and women. Because of differences in unadjusted PA scores between men and women across the waves, all analyses were repeated stratifying by sex (Table D in S1 File). To explore the effect of attrition on PA trajectories (which was mostly due to mortality; 47.6% participants died by 1 April 2012), the models were repeated in a sub-sample of 342 participants (118 men and 224 women) who had complete PA data (baseline and follow-ups) (Table E in S1 File).

## Results

### Characteristics of participants by level of physical activity at baseline

At baseline, 189 participants were classified as having low, 349 having medium, and 274 as having high PA (n = 812, 96.1% of the study sample) (Table 2). Thirty-three (3.91%) participants had no information about PA at baseline and were excluded from this analysis. Table B in S1 File shows the number of participants with available PA scores at each wave.

**Table 2:** Characteristics of participants by the levels of self-reported physical activity in the Newcastle 85+ study at baseline, n (%).

Characteristics	Low PA	Medium PA	High PA	All participants*	p
	189 (23.3)	349 (43.0)	274 (33.7)	812	
*Socio-demographic*, n (%)					
Sex					<0.001
men	65 (34.4)	101 (28.9)	145 (52.9)	311 (38.3)	
women	124 (65.6)	248 (71.1)	129 (47.1)	501 (61.7)	
Occupational class					0.08
routine	96 (56.8)	167 (50.5)	131 (48.7)	394 (51.2)	
intermediate	27 (16.0)	38 (11.5)	45 (16.7)	110 (14.3)	
higher managerial	46 (27.2)	126 (38.1)	93 (34.6)	265 (34.5)	
*Health-related*, n (%)					
Self-rated health					
excellent or very good	52 (29.2)	112 (32.6)	161 (59.4)	325 (41.0)	<0.001
good	65 (36.5)	149 (43.3)	81 (29.9)	295 (37.2)	
fair or poor	61 (34.3)	83 (24.1)	29 (10.7)	173 (21.8)	
GDS					
normal (0–5)	91 (61.5)	255 (74.6)	255 (93.4)	601 (78.8)	<0.001
mild (6–7)	31 (20.9)	55 (16.1)	12 (4.4)	98 (12.8)	
severe (8–15)	26 (17.6)	32 (9.4)	6 (2.2)	64 (8.4)	
Cognitive status (SMMSE)					<0.001
26–30 (normal)	83 (44.6)	263 (75.6)	236 (86.1)	582 (72.0)	
0–25 (impairment)	103 (55.4)	85 (24.4)	38 (13.9)	226 (28.0)	
Generalised pain past month					0.006
*no*	97 (54.2)	151 (43.5)	152 (55.5)	400 (50.0)	
*yes*	82 (45.8)	196 (56.5)	122 (44.5)	400 (50.0)	
Total prescribed medication					<0.001
0–2	13 (6.9)	45 (12.9)	75 (27.4)	133 (16.4)	
2–4	18 (9.5)	61 (17.5)	54 (19.7)	133 (16.4)	
≥5	158 (83.6)	243 (69.6)	145 (52.9)	546 (67.2)	
Disease count					<0.001
0–1	41 (22.0)	97 (27.8)	101 (36.9)	239 (29.4)	
2	48 (25.4)	98 (28.1)	98 (35.8)	244 (30.1)	
≥3	100 (52.9)	154 (44.1)	75 (27.4)	329 (40.5)	
*Anthropometry*					
Waist-hip ratio, M (SD)	0.9 (0.07)	0.88 (0.07)	0.88 (0.08)	0.88 (0.08)	0.005
*Lifestyle*					
Smoking status, n (%)					0.22
never smoker	72 (38.5)	129 (37.0)	85 (31.0)	286 (35.3)	
current smoker	12 (6.4)	22 (6.3)	12 (4.4)	46 (5.7)	
former smoker	103 (55.1)	198 (56.7)	177 (64.6)	478 (59.0)	
Current alcohol intake, n (%)					<0.001
no	107 (56.9)	150 (43.0)	69 (25.2)	326 (40.2)	
yes	81 (43.1)	199 (57.0)	205 (74.8)	485 (59.8)	
Energy from foods, M (SD)	6.81 (2.23)	6.59 (1.89)	7.25 (2.10)	6.87 (2.06)	<0.001

*All participants with baseline PA scores (96.1% of the cohort). PA, Physical Activity, SMMSE, Standardised Mini-Mental State Examination; GDS, Geriatric Depression Scale

Significance at α ≤ 0.05 for Gamma test for ordinal variables, and Fisher exact test or χ^2^ test for categorical variables, and Kruskal-Wallis test for non-normally distributed variables.

In unadjusted analysis, participants reporting high PA levels were more likely to be men, cognitively normal, report excellent or very good self-rated health, to have no depression, and to drink alcohol (all p<0.001). They were less likely to take ≥5 medications and to have ≥3 diseases compared to those in the low and medium PA groups (both p<0.001). Diseases that were less prevalent in participants with high PA levels were heart failure, cerebrovascular disease, osteoarthritis, osteoporosis, COPD, thyroid disease and diabetes (all p≤0.01) (Table B in S1 File). Other factors associated with lower PA levels were generalised pain lasting a day or more in the month prior to the study (p = 0.006) and waist-hip ratio (p = 0.005) (Table 2).

### Change in physical activity in the very old over 5 years (unadjusted analysis)

**Fig 1:**
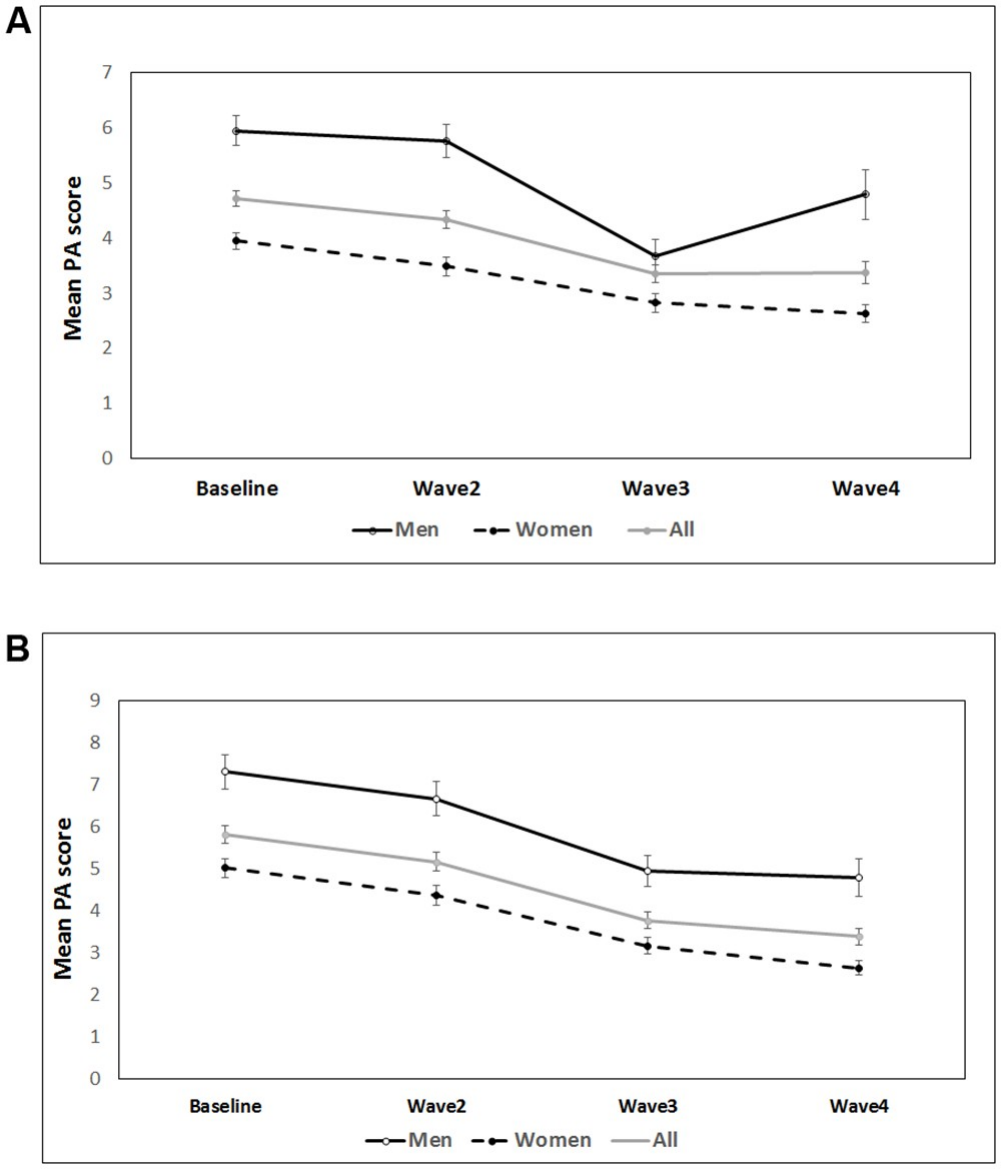
Self-reported physical activity (PA) scores in the Newcastle 85+ study. (A) Change in mean (unadjusted) PA scores in all participants (grey line), men (black solid line) and women (black dashed line) over 5 years. (B) Self-reported PA trajectory in 342 participants who completed all 4 PA assessments over 5 years of follow-up.

Throughout the study, men reported to being more active compared with women (i.e. higher unadjusted PA scores at each wave) (Table 3, Fig 1A).

**Table 3:** Self-reported physical activity scores (unadjusted) and physical activity levels over 5-year follow-up in the Newcastle 85+ study.

Waves	Men	n	Women	n	All	n	Low PA	Medium PA	High PA
	M (SD)		M (SD)		M (SD)		n (%)	n (%)	n (%)
Baseline	5.95 (4.76)	311	3.95 (3.35)	501	4.72 (4.07)	812	189 (23.3)	349 (43.0)	274 (33.7)
wave 2	5.76 (4.60)	234	3.49 (3.42)	395	4.34 (4.05)	629	167 (26.6)	274 (43.6)	188 (29.9)
wave 3	3.67 (4.16)	176	2.82 (3.08)	307	3.35 (3.58)	483	178 (36.9)	204 (42.2)	101 (20.9)
wave 4	4.79 (4.90)	118	2.63 (2.52)	226	3.37 (3.66)	344	114 (33.1)	164 (47.7)	66 (19.2)

PA, physical activity; low PA (score 0–1), medium PA (score 2–6), high PA (sore 7–18); wave 2 (1.5-year follow-up), wave 3 (3-year follow-up); wave 4 (5-year follow-up).

Fig 1 shows a non-linear change in PA scores over time, especially in men (panel A). Across all waves, the highest proportion of participants had medium levels of PA (42–48%), but there were increasing numbers of participants in low activity and decreasing numbers in high activity groups at subsequent waves (Table 3, Fig A in S1 File). More women than men reported low and medium PA over 5 years of follow-up (e.g. low PA at baseline, wave 2–4 in men: 20.9%, 19.7%, 34.1% and 30.5%, respectively versus 24.8%, 30.6%, 38.4%, and 34.5% in women; Fig A in S1 File). Conversely, at each wave a higher proportion of men were in the high PA group compared with women (Fig A in S1 File). In a sub-sample of 342 participants with complete PA assessments, we observed a deceleration of decline in unadjusted PA scores in all participants, and in men and women separately, especially between waves 3 and 4 (Fig 1B), that may be contributed to healthy survivor effect.

### Physical activity at baseline and 5-year trajectories and associated factors (adjusted analysis)

Table 4 reports the β (SE) estimates of the three LMM in all participants. We observed a significant linear (all p<0.001) and quadratic (all p<0.01) effect of time (i.e. decline in PA scores with deceleration in the rate of change over 5 years, probably due to mortality and healthy survivor effect). In the fully adjusted model, the following factors were independently associated with PA scores at baseline (Model 3): (a) sex (women had lower scores than men, mean difference of 1.91 points, p<0.001); (b) cognitive status (1.21 points higher in cognitively normal participants compared with cognitively impaired, p<0.01); (c) self-reported health (2.09 points higher in those reporting excellent / very good health than those in fair / poor health, p<0.001); (d) higher waist-hip ratio (5 points lower PA scores for every unit increase in ratio, p<0.01); (e) having ≤2 chronic diseases was associated with higher PA scores compared to having ≥3 diseases, and (f) not consuming alcohol was associated with lower PA scores (p<0.05). However, formal tests for interactions (confounder × time for cognitive status, self-rated health, disease, and sex) were not significant, indicating that the rate of change in PA scores over time did not vary by the baseline factors (details not shown).

**Table 4:** Parameter estimates^†^ of self-reported physical activity scores and associated factors in the Newcastle 85+ study over 5-year follow up.

Fixed effect	Model 1	Model 2	Model 3
	β (SE) ^†^	β (SE) ^†^	β (SE) ^†^
**PA intercept**	5.72 (0.30) ***	6.58 (1.93) ***	6.41 (2.05) **
Sex			
women	-2.09 (0.29)***	-2.16 (0.30)***	-1.91 (0.33)***
men	0	0	0
Occupational class			
routine/manual	-0.23 (0.30)	0.30 (0.28)	0.28 (0.28)
intermediate	1.17 (0.42)**	-0.74 (0.38)	0.63 (0.38)
higher managerial	0	0	0
Waist-hip ratio		-4.84 (1.89)*	-5.00 (1.90)**
Cognitive status			
≥26 SMMSE (normal)		1.28 (0.34)***	1.21 (0.36)**
0–25 SMMSE (impaired)		0	0
GDS			
no depression		0.74 (0.53)	0.73 (0.53)
mild		-0.64 (0.62)	-0.67 (0.63)
severe		0	0
Self-rated health			
excellent / very good		2.10 (0.39)***	2.09 (0.40)***
good		0.68 (0.38)	0.62 (0.39)
fair / poor		0	0
Disease count			
0–1		1.31 (0.34)***	1.25 (0.36)***
2		0.89 (0.30)**	0.87 (0.30)**
≥3		0	0
Total prescribed medication			
0–2		0.28 (0.36)	0.25 (0.36)
3–4		0.19 (0.34)	0.17 (0.34)
≥5		0	0
Generalised pain in the last month			
no		-0.04 (0.26)	-0.01 (0.26)
yes		0	0
Total energy from foods			0.07 (0.07)
Alcohol intake			
no			-0.58 (0.28)*
yes			0
Smoking status			
never smoker			0.03 (0.27)
current smoker			-0.16 (0.61)
former smoker			0
**PA decline**			
Time (years)	-0.47 (0.12)***	-0.53 (0.13)***	-0.52 (0.13)***
Time^2^	0.07 (0.02)**	0.08 (0.02)**	0.07 (0.02)**
Goodness of fit statistics			
Akaike information criterion (AIC)	8633.03	7896.28	7815.69

*** p<0.001;

** p<0.01;

* p<0.05.

^†^β coefficients (SE) are estimates of fixed effects with longitudinal PA data to evaluate the population averages in PA using LLM. Fixed effects for covariates estimated initial level and trajectory differences in PA as a function of the covariate in the model. The main effect of time (time and time^2^) tested linear change in PA scores and acceleration / deceleration in PA change over 5 years, respectively.

Model 1 includes time (linear and quadratic) and socio-demographic variables.

Model 2 is additionally adjusted for health-related variables (waist-hip ratio, cognitive status, depressive symptoms, self-rated health, number of chronic diseases, number of medication, and pain in the last month).

Model 3 is further adjusted for lifestyle factors (total energy from foods, current alcohol intake, and smoking status).

LMM, linear mixed model; GDS, Geriatric Depression Scale; PA, physical activity; SMMSE, Standardized Mini Mental State Examination.

In stratified analyses, better cognitive status and self-rated health were significantly associated with higher PA scores in both men and women (Table D in S1 File). However, waist-hip ratio, mild depression, and number of chronic diseases and medications were associated with PA scores at baseline only in women (Table D in S1 File). Table E in S1 File reports the β (SE) estimates of the three LMM in participants with complete PA data. We observed a significant linear (all p≤0.002) and quadratic (all p≤0.004) effect of time (i.e. a non-linear decline in PA scores over 5 years). In the fully adjusted model, factors significantly associated with PA scores at baseline were female gender (-2.26 (0.40), p<0.001), excellent / very good self-rated health (2.12 (0.52), p<0.001), and lower disease count (0–1 diseases: 1.48 (0.42), p = 0.01) compared to the referent group.

## Discussion

The current demographic trends show a fast rise in the number of people aged 85+ (the very old) (a 351% estimated increase from 2010–2050 globally [38]). There is a need for better understanding about how PA as a non-genetic risk factor changes over time and influences the ageing process beyond the young-old age. Utilising data from the Newcastle 85+ Study we have shown that medium levels of PA (i.e. a score of 2–6 on PA questionnaire ranging from 0–18) were most commonly reported at each assessment over the 5-year follow-up. Those who reported high levels of PA at baseline (score 7–18) were more likely to be men, to be healthier (fewer chronic diseases and no history of depression), and to drink alcohol. PA declined by 0.5 points/year in all participants but the fall was not linear and decelerated by 0.07 points/year over the study period. This may be explained by a higher proportion of men compared with women who survived between 3- and 5-years follow-up (from ages 88 to 90) and reported taking part in very and moderately energetic activities 1–3 times a week compared with women (e.g. at wave 4: 35.6% (n = 42) of men were engaged in these activities compared with 10.2% (n = 23) women). Factors significantly associated with PA scores were gender, waist-hip ratio, cognitive status, self-rated health, number of chronic diseases, and alcohol consumption. However, the rate of decline in PA (slopes) did not vary by any of the factors over time in all participants and in those whose PA was assessed at each wave over 5 years. Although men and women differed at each PA assessment (higher PA scores in men), their trajectories of change in PA were similar.

To date, several studies have addressed long-term changes in self-reported PA in older adults aged 65+ or in those transitioning to old age by describing either their activity trajectory classes (patterns of change) [14,17,25] or examining the rate of change in PA (average trend in PA) [15,16,18–20,22,39]. The comparison between the results from these and our study is limited because of differences in length of follow-up, PA questionnaires used to assess the type, duration, intensity and frequency of self-reported PA, and a low number of the very old included in the studies [14,19,22,39]. Overall, previous studies showed a general decline in PA [15,18–20,39] with age in both men and women, and an increase in inactivity / sedentary behaviour (e.g. [16,22]), although patterns of increased PA and stability in PA (e.g. [14,17,18]) have also been reported. This suggests heterogeneity in PA and PA trends in older adults over time, which may be influenced by factors other than age and gender. For example, the English Longitudinal Study of Ageing of over 5,000 older adults (mean age 61) has used a similar PA questionnaire to the Newcastle 85+ Study, and found an overall trend for increase in inactivity and decrease in vigorous PA over a 10-year follow-up [22]. However, about 50% of the sample was classified as persistently active (i.e. having moderate and / or vigorous PA at least once a week over the study period). Advanced age, female gender, long-standing illness, depression, arthritis, obesity, and ever smoking were associated with lower odds of being persistently active. About 50% of older adults (aged 65+) in the ‘Aging in Chianti’ study experienced no change in average PA (measured on a 6-point scale) and about 13% reported an increase in PA over 3-year follow-up [16]. About 17% of the Taiwanese older adults (over 4,000 participants aged 50 to 96) remained active, and about 23% had an increased PA over 11-year follow-up [14]. In the Health and Retirement Study, a national sample of over 7,500 American adults (aged 54–72), the odds of participation in regular (vigorous) PA declined by about 4% per year over 6-year follow-up, and was steeper in less educated participants [18]. Physical leisure activity declined linearly and accelerated after the age of 70 years in the Swedish / Adoption Twin Study of Aging of adults aged 36–91, especially in older women [39].

Little is known about PA change and its determinants in the very old, and whether they differ across genders. In the present study, most participants (both men and women) reported moderate levels of PA, such as walking at moderate pace, moderate gardening and heavy housework across 5 years of follow-up, suggesting a stability in the number of the very old reporting moderate PA levels (Fig A in S1 File). Walking, indoor household tasks and gardening have been reported as the most common PA activities in older adults belonging to young-old [17,24,40–42]. In the New York City Neighborhood and Mental Health in the Elderly Study II of 3,500 older residents of New York (aged 65–75) about 90–92% reported ever walking (about half of them at least 30 minutes/day), 53–55% heavy housework and 19–21% gardening across 3-year follow-up without major transitions in habitual patterns comprised of these activities [42]. Interventions aimed to promote and maintain PA in older adults [43] should consider activities that show stability in very old age, which will allow for better understanding of related influences that prevent maintenance of PA over time.

As expected, PA declined on average by a 1/2 point per year in the very old, but decelerated slightly across time. A higher percentage of men had high PA levels across the waves, whilst women’s high PA levels declined more (15% versus 11% from baseline to wave 4; Fig A in S1 File), and more women than men reported medium PA over 5 years (Fig A in S1 File). This could be explained by a survivor effect and the ability of a subgroup of older men to maintain higher levels of PA over time because of higher physiological reserve, fewer diseases and functional limitations compared with women [44]. Strategies in designing PA interventions for the very old who sustain their level of PA may differ from the strategies aimed to increase activity levels in the very old who have low activity or are inactive. Additionally, future research focused on those who maintain or increase levels of PA in very late life may be informative for developing beneficial interventions for at risk groups for PA decline such as highly active very old women.

Factors such as health status (i.e. higher burden of chronic diseases in women; depression; polypharmacy), lower self-perceived health, no alcohol intake and higher waist-hip ratio identified in this cohort to be negatively associated with PA scores should be regarded in PA interventions in this age group, especially those that are modifiable. Similar to our findings, previous studies have reported worse overall health, long-standing chronic diseases, and depression as predictors of low PA in older adults aged 65+ [17,22–26]. Whilst PA promotes mental health and wellbeing in older adults [45], depressive symptoms may act as a barrier to sustained PA and should be targeted in clinical practice and policy [22,26,45].

None of the factors were associated with the rate of PA decline, which could be explained by the loss of power in data. By wave 4, 57.6% of participants with established PA scores at baseline had been lost to follow-up, which was mostly due to mortality.

As reported in a number of studies (e.g. [14–21]), women had lower PA scores (2 point lower overall score) compared with men, which may be partially explained by higher disease burden and consequent greater functional limitations in women [44]. An inverse relationship between chronic diseases and sufficient leisure-time PA has been reported in the National Health Interview Survey of over 36,700 adults; every additional disease decreased the odds of reaching ≥150 minutes/week of PA by 17%. In older women aged 70–79, every additional chronic disease was associated with a 70% increased odds of being ‘always inactive’ compared with ‘always active’ PA pattern in the Women’s Health and Aging Study [17]. Future studies should explore the bidirectional relationship between PA and chronic diseases in older women. Whilst maintaining higher PA has been linked to a reduced risk of several chronic diseases, higher disease burden has been consistently reported as a significant predictor of low PA levels and PA decline [46].

Another explanation for higher levels of PA in men could be contributed to by the PA domains assessed in the questionnaire that are more relevant to men. At each wave, women were more likely to say that they hardly ever or never engaged in highly energetic activities such as swimming, cycling, running, heavy gardening (all p<0.001; Table A in S1 File) whilst there were no differences in reporting mildly energetic activities (light housework and light gardening) between the genders. Previous research has reported that determinants and change in PA are domain-specific [20], which has not been established beyond the age of 85.

Current alcohol intake was positively associated with PA scores. Other studies have also reported a positive association between moderate alcohol intake and PA [25,47], which could be explained by greater social connections related to alcohol intake [47], and beneficial cardiovascular outcomes associated with moderate alcohol consumption. Future investigations should determine whether moderate alcohol intake moderates the beneficial cardiovascular outcome related with PA; and whether PA moderates cardiovascular effects associated with alcohol intake in older adults.

### Study strengths and limitations

The strengths of our study include: (a) longitudinal design; (b) representativeness of the very old population in the UK despite being a moderately sized cohort; (c) growth curve modelling which took into account varying intercepts and slopes of PA and missing data, and described within-individual change over time and related predictors of inter-individual differences in change; (d) a purpose-designed PA questionnaire that was based on the results from the Newcastle 85+ Pilot Study [33], and (e) previously established convergent validity of self-reported PA with several domains of objective measures of PA (accelerometry) in this cohort [35]. Despite these strengths, the following has to be considered when interpreting the results: (a) although the Newcastle 85+ PA questionnaire demonstrated reasonable convergent validity with objective measures [35], self-reports are subject to bias and misclassification and are dependent on recall; (b) the results have limited generalisability to other cohorts of the very old, particularly from different ethnic backgrounds, but because of the representativeness of the cohort, may be generalisable to many very old adults living in other regions of the UK; (c) as with any cohort of 85-year olds, loss to follow-up was high (mortality), which may have underestimated β coefficients in the models, and contributed to selection bias, and (d) although we explored a range of confounders and considered model fit statistics, unexplained confounding (e.g. social networks, self-efficacy, sedentary behaviour, vision and hearing loss, incontinence, and fear of falling) may have affected β estimates for each confounder.

### Conclusion

In summary, sustaining higher physical activity is known to play an important role for physical, mental health, functioning and independence in older adults aged 65 years and over. However to date, there has been relatively little focus on understanding influences of PA change in very old adults aged 85 years and over despite this being the fastest growing age group of the adult UK population. In this study of the very old, we have found that not all older adults experience decline in self-reported PA—stability in moderate and increases in high PA have been also observed. Factors associated with PA scores were gender, number of chronic diseases, self-rated health and cognitive health, waist-hip ratio, and alcohol consumption. However, none of these factors were associated with the rate of change in PA over 5 years. Future research focused on the very old who maintain or increase levels of PA in very late life may be informative for developing beneficial interventions for at risk groups for PA decline.

## Supporting information

S1 File Fig A. Self-reported physical activity levels in women, men and all participants from the Newcastle 85+ study. Table A. Frequency of very energetic, moderately energetic and mildly energetic physical activity in all participants, men and women in the Newcastle 85+ study. Table B. Number of participants and pattern of missing self-reported physical activity scores in the Newcastle 85+ Study over 5-year follow up. Table C. Numbers with (prevalence of) individual chronic diseases at baseline by the levels of physical activity in the Newcastle 85+ Study, % (n). Table D. Parameter estimates for self-reported physical activity scores and associated factors in men and women in the Newcastle 85+ Study over 5-year follow up. Table E. Parameter estimates for self-reported physical activity scores and associated factors in participants with complete physical activity data in the Newcastle 85+ Study over 5-year follow up.
(DOCX)
