# Supplementary material for: Factors associated with change in self-reported physical activity in the very old: The Newcastle 85+ study
Source: PLoS One. 2019 Jul 16;14(7):e0218881. doi: 10.1371/journal.pone.0218881 (PMC6634376; doi:10.1371/journal.pone.0218881)
Supplement: S1 File — Fig A. Self-reported physical activity levels in women, men and all participants from the Newcastle 85+ study. Table A. Frequency of very energetic, moderately energetic and mildly energetic physical activity in all participants, men and women in the Newcastle 85+ study. Table B. Number of participants and pattern of missing self-reported physical activity scores in the Newcastle 85+ Study over 5-year follow up. Table C. Numbers with (prevalence of) individual chronic diseases at baseline by the levels of physical activity in the Newcastle 85+ Study, % (n). Table D. Parameter estimates for self-reported physical activity scores and associated factors in men and women in the Newcastle 85+ Study over 5-year follow up. Table E. Parameter estimates for self-reported physical activity scores and associated factors in participants with complete physical activity data in the Newcastle 85+ Study over 5-year follow up. (DOCX) [file pone.0218881.s001.docx]

**S1 File: Supporting Information**

**
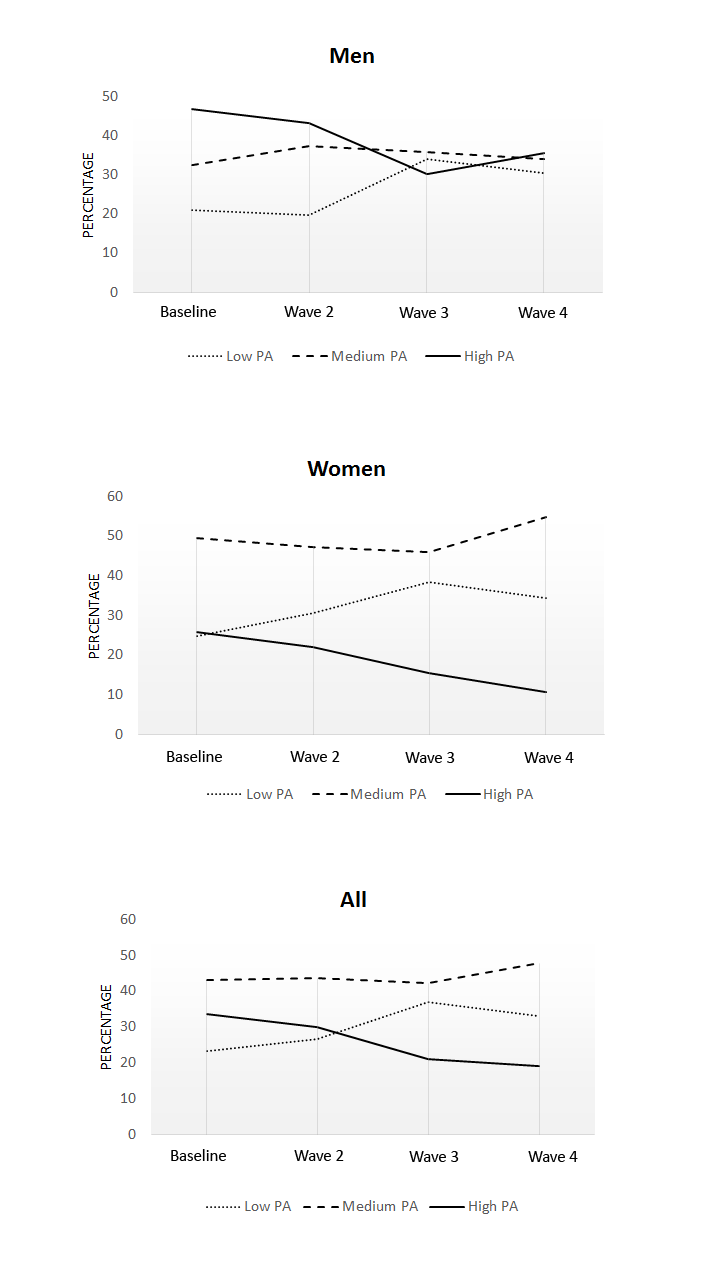
**

**Fig A. Self-reported physical activity levels in women, men and all participants from the Newcastle 85+ Study.** Low PA levels (dotted black line) increased, whilst high PA (solid black line) decreased over the 5-year follow-up (from baseline to wave 4). Medium PA levels (dashed black line) remained stable and slightly increased from wave 3 to wave 4 in all participants and women.

**Table A.** **Frequency of very energetic, moderately energetic and mildly energetic physical activity in all participants, men and women in the Newcastle 85+ Study**

| **Wave** | **Intensity** | **Frequency** | **All** | **Men** | **Women** | **p^*^** |
| --- | --- | --- | --- | --- | --- | --- |
|  |  |  | n (%) | n (%) | n (%) |  |
| Baseline | Very energetic | ≥3 times/week | 15 (1.8) | 14 (93.3) | 1 (6.7) | <0.001 |
|  |  | 1-2 times/week | 28 (3.4) | 21 (75.0) | 7 (25.0) |  |
|  |  | 1-3 times/month | 35 (4.3) | 25 (71.4) | 10 (28.6) |  |
|  |  | hardly ever/never | 735 (90.4) | 251 (34.1) | 484 (65.9) |  |
|  | Moderately energetic | ≥3 times/week | 179 (21.9) | 90 (50.6) | 88 (49.4) | <0.001 |
|  |  | 1-2 times/week | 106 (13.0) | 58 (54.7) | 48 (45.3) |  |
|  |  | 1-3 times/month | 95 (11.7) | 36 (37.9) | 59 (62.1) |  |
|  |  | hardly ever/never | 434 (53.4) | 127 (29.3) | 307 (70.7) |  |
|  | Mildly energetic | ≥3 times/week | 494 (60.8) | 189 (38.3) | 305 (61.7) | 0.45 |
|  |  | 1-2 times/week | 111 (13.7) | 48 (43.2) | 63 (56.8) |  |
|  |  | 1-3 times/month | 38 (4.7) | 16 (42.1) | 22 (57.9) |  |
|  |  | hardly ever/never | 170 (20.9) | 58 34.1) | 112 (65.9) |  |
| Wave2 | Very energetic | ≥3 times/week | 9 (1.4) | 6 (66.7) | 3 (33.3) | <0.001 |
|  |  | 1-2 times/week | 24 (3.8) | 20 (83.3) | 4 (16.7) |  |
|  |  | 1-3 times/month | 21 (3.3) | 14 (66.7) | 7 (33.3) |  |
|  |  | hardly ever/never | 575 (91.4) | 194 (33.7) | 381 (66.3) |  |
|  | Moderately energetic | ≥3 times/week | 119 (18.9) | 69 (58.0) | 50 (42.0) | <0.001 |
|  |  | 1-2 times/week | 77 (12.2) | 35 (45.5) | 42 (54.5) |  |
|  |  | 1-3 times/month | 62 (9.9) | 28 (45.2) | 34 (54.8) |  |
|  |  | hardly ever/never | 371 (59.0) | 102 (27.5) | 269 (72.5) |  |
|  | Mildly energetic | ≥3 times/week | 345 (54.8) | 142 (41.2) | 203 (58.8) | <0.001 |
|  |  | 1-2 times/week | 106 (16.9) | 40 (37.7) | 66 (62.3) |  |
|  |  | 1-3 times/month | 48 (7.6) | 17 (35.4) | 31 (64.6) |  |
|  |  | hardly ever/never | 130 (20.7) | 35 (26.9) | 95 (73.1) |  |
| Wave3 | Very energetic | ≥3 times/week | 5 (1.0) | 3 (60.0) | 2 (40.0) | <0.001 |
|  |  | 1-2 times/week | 6 (1.2) | 5 (83.3) | 1 (16.7) |  |
|  |  | 1-3 times/month | 14 (2.9) | 12 (85.7) | 2 (14.3) |  |
|  |  | hardly ever/never | 458 (94.8) | 156 (34.1) | 302 (65.9) |  |
|  | Moderately energetic | ≥3 times/week | 69 (14.3) | 37 (53.6) | 32 (46.4) | <0.001 |
|  |  | 1-2 times/week | 34 (7.0) | 15 (44.1) | 19 (55.9) |  |
|  |  | 1-3 times/month | 41 (8.5) | 24 (58.5) | 17 (41.5) |  |
|  |  | hardly ever/never | 339 (70.2) | 100 (29.5) | 239 (70.5) |  |
|  | Mildly energetic | ≥3 times/week | 216 (44.7) | 83 (38.4) | 133 (61.6) | 0.91 |
|  |  | 1-2 times/week | 86 (17.8) | 30 (34.9) | 56 (65.1) |  |
|  |  | 1-3 times/month | 44 (9.1) | 22 (50.0) | 22 (50.0) |  |
|  |  | hardly ever/never | 137 (28.4) | 41 (29.9) | 96 (70.1) |  |
| Wave4 | Very energetic | ≥3 times/week | 6 (1.7) | 6 (100.0) | 0 (0.0) | <0.001 |
|  |  | 1-2 times/week | 3 (0.9) | 3 (100.0) | 0 (0.0) |  |
|  |  | 1-3 times/month | 9 (2.6) | 6 (66.7) | 3 (33.3) |  |
|  |  | hardly ever/never | 326 (94.8) | 103 (31.6) | 223 (68.4) |  |
|  | Moderately energetic | ≥3 times/week | 43 (12.5) | 26 (60.5) | 17 (39.5) | <0.001 |
|  |  | 1-2 times/week | 23 (6.7) | 17 (73.9) | 6 (26.1) |  |
|  |  | 1-3 times/month | 21 (6.1) | 7 (33.3) | 14 (66.7) |  |
|  |  | hardly ever/never | 257 (74.7) | 68 (26.5) | 189 (73.5) |  |
|  | Mildly energetic | ≥3 times/week | 206 (59.9) | 73 (35.4) | 133 (64.6) | 0.92 |
|  |  | 1-2 times/week | 19 (5.5) | 7 (36.8) | 12 (63.2) |  |
|  |  | 1-3 times/month | 13 (3.8) | 4 (30.8) | 9 (69.2) |  |
|  |  | hardly ever/never | 106 (30.8) | 34 (32.1) | 72 (67.9) |  |

*Chi-square test.

**Table B. Number of participants and pattern of missing self-reported physical activity scores in the Newcastle 85+ Study over 5-year follow up**

| **n** | **wave 1** | **wave 2** | **wave 3** | **wave 4** |
| --- | --- | --- | --- | --- |
| Complete PA scores |  |  |  |  |
| 342 | 1 | 1 | 1 | 1 |
| Incomplete PA scores |  |  |  |  |
| 140 | 1 | 1 | 1 | 0 |
| 145 | 1 | 1 | 0 | 0 |
| 184 | 1 | 0 | 0 | 0 |
| 32 | 0 | 0 | 0 | 0 |

In the pattern of missing data, 1 stands for available PA score and 0 otherwise.

Wave 1 (baseline), wave 2 (1.5-year follow-up), wave 3 (3-year follow-up); wave 4 (5-year follow-up).

**Table C.** **Numbers with (prevalence of) individual chronic diseases at baseline by the levels of physical activity in the Newcastle 85+ Study, % (n)**

| **Disease** | **Low PA, % (n)** | **Medium PA, % (n)** | **High PA, % (n)** | **All, % (n)** | **p** |
| --- | --- | --- | --- | --- | --- |
| Hypertension | 59.3 (112) | 57.3 (200) | 55.1 (151) | 57.0 (463) | 0.67 |
| Ischaemic heart | 39.4 (71) | 37.5 (128) | 31.7 (86) | 36.0 (285) | 0.18 |
| Heart failure | 18.0 (34) | 12.3 (43) | 6.9 (19) | 11.8 (96) | 0.001 |
| Atrial fibrillation or flutter | 17.7 (31) | 14.5 (49) | 11.5 (31) | 14.2 (111) | 0.18 |
| Cerebrovascular disease | 32.3 (61) | 21.8 (76) | 11.7 (32) | 20.8 (169) | <0.001 |
| Peripheral vascular disease | 10.6 (20) | 6.6 (23) | 5.1 (14) | 7.0 (57) | 0.07 |
| All osteoarthritis-related diseases | 56.6 (107) | 60.7 (212) | 49.6 (136) | 56.0 (455) | 0.02 |
| Inflammatory arthritis | 5.3 (10) | 4.9 (17) | 2.2 (6) | 4.1 (33) | 0.15 |
| Osteoporosis | 19.6 (37) | 13.8 (48) | 8.0 (22) | 13.2 (107) | 0.001 |
| COPD | 19.6 (37) | 19.5 (68) | 11.0 (30) | 16.6 (135) | 0.008 |
| Asthma | 6.4 (12) | 2.9 (10) | 4.4 (12) | 4.2 (34) | 0.15 |
| Thyroid diseases | 16.9 (32) | 18.6 (65) | 8.0 (22) | 14.7 (119) | 0.001 |
| Diabetes | 20.6 (39) | 13.8 (48) | 9.1 (25) | 13.8 (112) | 0.002 |
| Cancer^*^ | 5.3 (10) | 8.3 (29) | 4.7 (13) | 6.4 (52) | 0.15 |
| Renal impairment | 28.7 (49) | 23.9 (79) | 20.8 (56) | 23.9 (184) | 0.17 |

^*^Excluding skin carcinoma and solar keratosis in the past 5 years; COPD, chronic obstructive pulmonary disease.

**Table D.** **Parameter estimates^†^ for self-reported physical activity scores and associated factors in men and women in the Newcastle 85+ Study over 5-year follow up**

| **Fixed Effect** | **Model 1** | **Model 2** | **Model 3** | **Model 1** | **Model 2** | **Model 3** |
| --- | --- | --- | --- | --- | --- | --- |
|  | β (SE)^†^ | β (SE)^†^ | β (SE)^†^ | β (SE)^†^ | β (SE)^†^ | β (SE)^†^ |
|  | **Men** |  |  | **Women** |  |  |
| **PA intercept** | 5.88 (0.48)*** | 3.24 (3.93) | 2.58 (4.16) | 3.51 (0.28)*** | 5.68 (1.83)** | 5.74 (1.95)** |
| Occupations class |  |  |  |  |  |  |
| routine / manual | -0.59 (0.58) | 0.03 (0.57) | -0.14 (0.58) | -0.005 (0.32) | 0.47 (0.28) | 0.45 (0.28) |
| intermediate | 2.62 (1.15)* | 1.82 (1.06) | 1.28 (1.07) | 0.92 (0.40)* | 0.44 (0.33) | 0.40 (0.34) |
| higher managerial | 0 | 0 | 0 | 0 | 0 | 0 |
| Waist-hip ratio |  | -3.56 (3.83) | -3.18 (3.83) |  | -5.62 (1.94)** | -5.85 (1.97)** |
| Cognitive status |  |  |  |  |  |  |
| ≥26 SMMSE |  | 2.14 (0.68)** | 1.86 (0.69)** |  | 1.03 (0.35)** | 1.06 (0.36)** |
| 0-25 SMMSE |  | 0 | 0 |  | 0 | 0 |
| GDS |  |  |  |  |  |  |
| no depression |  | 1.98 (1.25) | 2.27 (1.25) |  | 0.24 (0.48) | 0.18 (0.49) |
| mild |  | 1.00 (1.59) | 1.01 (1.62) |  | -1.09 (0.56) | -1.15 (0.57)* |
| severe |  | 0 | 0 |  | 0 | 0 |
| Self-rated health |  |  |  |  |  |  |
| excellent/very good |  | 3.07 (0.98)** | 2.96 (0.99)** |  | 1.66 (0.37)*** | 1.64 (0.37)*** |
| good |  | 0.77 (0.97) | 0.56 (0.97) |  | 0.79 (0.35)* | 0.75 (0.36)* |
| fair/poor |  | 0 | 0 |  | 0 | 0 |
| Disease count |  |  |  |  |  |  |
| 0-1 |  | 0.96 (0.72) | 0.79 (0.73) |  | 1.29 (0.34)*** | 1.30 (0.34)*** |
| 2 |  | 0.45 (0.63) | 0.41 (0.64) |  | 1.13 (0.30) | 0.75 (0.36)* |
| ≥3 |  | 0 | 0 |  | 0 | 0 |
| Total medication |  |  |  |  |  |  |
| 0-2 |  | -0.50 (0.74) | -0.66 (0.75) |  | 0.97 (0.37)** | 1.00 (0.38)** |
| 3-4 |  | 0.59 (0.70) | 0.58 (0.70) |  | -0.02 (0.33) | -0.02 (0.34) |
| ≥5 |  | 0 | 0 |  | 0 | 0 |
| Generalised pain |  |  |  |  |  |  |
| no |  | 0.28 (0.55) | 0.31 (0.56) |  | -0.09 (0.25) | -0.04 (0.26) |
| yes |  | 0 | 0 |  |  | 0 |
| Energy |  |  | 0.13 (0.12) |  |  | 0.03 (0.08) |
| Alcohol intake |  |  |  |  |  |  |
| no |  |  | -1.68 (0.74)* |  |  | -0.26 (0.25) |
| yes |  |  | 0 |  |  | 0 |
| Smoking status |  |  |  |  |  |  |
| never smoker |  |  | -0.32 (0.59) |  |  | 0.15 (0.25) |
| current smoker |  |  | 0.03 (1.54) |  |  | -0.09 (0.57) |
| former smoker |  |  | 0 |  |  | 0 |
| **PA decline** |  |  |  |  |  |  |
| Time (years) | -0.60 (0.23)** | -0.70 (0.24)** | -0.73 (0.24)** | -0.40 (0.13)* | -0.44 (0.14)** | -0.41 (0.14)** |
| Time^2^ | 0.09 (0.04)* | 0.11 (0.05)* | 0.11 (0.5)* | 0.05 (0.02)* | 0.06 (0.03)* | 0.05 (0.03)* |
| Goodness of fit |  |  |  |  |  |  |
| AIC | 3337.66 | 3082.11 | 3043.79 | 5564.62 | 4685.22 | 4644.75 |

*** p<0.001; ** p<0.01; * p<0.05.

^†^β coefficients (SE) are estimates of fixed effects with longitudinal PA data to evaluate the population averages in PA using LLM. Fixed effects for covariates estimated initial level and trajectory differences in PA as a function of the covariate in the model. The main effect of time (time and time2) tested linear change in PA scores and acceleration/deceleration in PA change over 5 years, respectively.

Model 1 includes time (linear and quadratic) and occupational class.

Model 2 is additionally adjusted for health-related variables (waist-hip ratio, cognitive status, depressive symptoms, self-rated health, number of chronic diseases, number of medication, and pain in the last month).

Model 3 is further adjusted for lifestyle factors (total energy from foods, current alcohol intake, and smoking status).

LMM, linear mixed model; GDS, Geriatric Depression Scale; PA, physical activity; SMMSE, Standardized Mini Mental State Examination; AIC, Akaike Information Criterion.

**Table E. Parameter estimates^†^ for self-reported physical activity scores and associated factors in participants with complete physical activity data in the Newcastle 85+ Study over 5-year follow up**

| **Fixed Effect** | **Model 1** | **Model 2** | **Model 3** |
| --- | --- | --- | --- |
|  | β (SE)^a^ | β (SE)^a^ | β (SE)^a^ |
| **PA intercept** | 6.49 (0.36) *** | 6.40 (2.38) ** | 6.03 (2.52) * |
| Sex |  |  |  |
| women | -2.23 (0.36)*** | -2.42 (0.36)*** | -2.26 (0.40)*** |
| men | 0 | 0 | 0 |
| Occupations class |  |  |  |
| routine / manual | 0.12 (0.37) | 0.64 (0.34) | 0.60 (0.34) |
| intermediate | 1.16 (0.50) | 0.84 (0.44) | 0.73 (0.45) |
| higher managerial | 0 | 0 | 0 |
| Waist-hip ratio |  | -3.86 (2.33) | -3.85 (2.36) |
| Cognitive status |  |  |  |
| ≥26 SMMSE (normal) |  | 0.79 (0.43)*** | 0.83 (0.44) |
| 0-25 SMMSE (impaired) |  | 0 | 0 |
| GDS |  |  |  |
| no depression |  | 1.14 (0.66) | 1.14 (0.67) |
| mild |  | -0.97 (0.80) | -0.98 (0.80) |
| severe |  | 0 | 0 |
| Self-rated health |  |  |  |
| excellent/very good |  | 2.05 (0.51)*** | 2.12 (0.52)*** |
| good |  | 0.23 (0.50) | 0.22 (0.51) |
| fair/poor |  | 0 | 0 |
| Disease count |  |  |  |
| 0-1 |  | 1.51 (0.42)*** | 1.48 (0.42)*** |
| 2 |  | 0.91 (0.37)* | 0.88 (0.37)* |
| ≥3 |  | 0 | 0 |
| Total prescribed medication |  |  |  |
| 0-2 |  | 0.38 (0.43) | 0.34 (0.43) |
| 3-4 |  | 0.16 (0.40) | 0.12 (0.40) |
| ≥5 |  | 0 | 0 |
| Generalised pain in the last month |  |  |  |
| no |  | 0.07 (0.31) | 0.08 (0.31) |
| yes |  | 0 | 0 |
| Total energy from foods |  |  | 0.05 (0.09) |
| Alcohol intake |  |  |  |
| no |  |  | -0.40 (0.35) |
| yes |  |  | 0 |
| Smoking status |  |  |  |
| never smoker |  |  | 0.05 (0.32) |
| current smoker |  |  | 0.18 (0.85) |
| former smoker |  |  | 0 |
| **PA decline** |  |  |  |
| Time (years) | -0.51 (0.15)*** | -0.51 (0.15)*** | -0.49 (0.16)** |
| Time^2^ | 0.09 (0.03)*** | 0.09 (0.03)** | 0.09 (0.03)** |

***p<0.001; **p<0.01; *p<0.05.

^†^β coefficients (SE) are estimates of fixed effects with longitudinal PA data to evaluate the population averages in PA using LLM. Fixed effects for covariates estimated initial level and trajectory differences in PA as a function of the covariate in the model. The main effect of time (time and time2) tested linear change in PA scores and acceleration/deceleration in PA change over 5 years, respectively.

Model 1 includes time (linear and quadratic) and occupational class.

Model 2 is additionally adjusted for health-related variables (waist-hip ratio, cognitive status, depressive symptoms, self-rated health, number of chronic diseases, number of medication, and pain in the last month).

Model 3 is further adjusted for lifestyle factors (total energy from foods, current alcohol intake, and smoking status).

LMM, linear mixed model; GDS, Geriatric Depression Scale; PA, physical activity; SMMSE, Standardized Mini Mental State Examination.
